# Proteoglycans in Breast Cancer: Friends and Foes

**DOI:** 10.3390/biom15121688

**Published:** 2025-12-03

**Authors:** Noelia Vigo-Díaz, Rubén López-Cortés, Isabel Velo-Heleno, Laura Rodríguez-Silva, Cristina Núñez

**Affiliations:** 1 Inorganic Chemistry Department, Faculty of Sciences, Campus Terra, University of Santiago de Compostela, 27002 Lugo, Spain; noelia.vigo@usc.es (N.V.-D.); laura.rodriguez@usc.es (L.R.-S.); 2 Research Unit, Hospital Universitario Lucus Augusti (HULA), Servizo Galego de Saúde (SERGAS), 27003 Lugo, Spain; ruben.lopez.cortes@sergas.es; 3 Inorganic Chemistry Department, Faculty of Chemistry, University of Santiago de Compostela, 15782 Santiago de Compostela, Spain; mariaisabel.velo.heleno@usc.es

**Keywords:** proteoglycans, breast cancer, extracellular matrix remodeling, tumour microenvironment, biomarkers, precision medicine

## Abstract

Proteoglycans (PGs) are highly glycosylated proteins of great importance both structurally and for signalling in the extracellular matrix (ECM) as well as cell surfaces. In breast cancer (BC), they control the structure of tissue architecture, cellular communication pathways and tumour–stroma interactions, thus affecting adhesion, migration, angiogenesis, immune evasion, and metastasis. Their structural heterogeneity supports either subtype- or context-dependent functions. This review combines current studies of PGs in BC according to their classification into intracellular, cell-surface, pericellular, extracellular, and small leucine-rich PGs and a range of non-classical PGs. A literature-driven approach to focus on molecular mechanisms and clinical correlations will demonstrate how PGs respond with collagens, growth factors, cytokines, and proteolytic enzymes in order to modulate the ECM and affect therapy resistance. Indeed, PGs including syndecans, glypicans, perlecan, versican, biglycan and decorin showed the potential to be promoters or suppressors of cancer, with local effects on invasion, and have a significant modulating effect on BC subtypes or the prognosis and therapeutic response and may potentially serve as new biomarkers for stratification and liquid biopsy candidates. Furthermore, PGs appear to modulate the tumour immune landscape, are involved in the development of metastatic niches, and underlie signalling pathways like Wnt or TGFβ in a subtype-dependent manner, extending their translational prospects and therapeutic utility. PGs, taken together, seem to be major modulators of BC, with particular relevance for precision medicine.

## 1. Introduction

PGs are crucial elements in the ECM and on the cell surface and they are involved in the tissue structure and cell signalling [1]. They are formed by adhesion of a core protein to glycosaminoglycan (GAG) chains: heparan sulfate (HS), chondroitin sulfate (CS), dermatan sulfate (DS), keratan sulfate (KS), and hyaluronic acid (HA). In BC, PGs regulate key processes involved in tumour progression, such as cell adhesion, migration, and invasion, and participate in additional signaling pathways [2]. As an example, syndecan-1, a cell-surface heparan sulfate proteoglycan (HSPG), correlates with aggressive features in BC. Its expression is correlated with tumour invasiveness and poor prognosis, underlining its promise as a therapeutic target [3]. Moreover, perlecan, which is a basement membrane (BM) PG, regulates ECM stiffness and stimulates angiogenesis, driving tumour growth and metastasis [4]. These PGs influence cell response, interact with and modulate growth factors and cytokines, and modulate the ECM dynamic remodelling in the tumour microenvironment (TME). Knowledge of the complex roles of PGs in BC will help to guide the design of directed treatments targeting ECM modulations that may suppress BC progression. During BC, the ECM becomes highly altered, and PGs are pivotal in this process. Tumour cells and stromal fibroblasts secrete PGs that bind to ECM proteins, affecting tissue stiffness and cellular behaviour. For instance, small leucine-rich PG, decorin, affects collagen fibrillogenesis and suppresses angiogenesis, thus limiting tumour growth. In contrast, versican and biglycan PGs, other matrix-secreted PGs, accumulate in the peritumoural ECM, stimulate inflammatory pathways, and drive tumour invasion and early relapse in node-negative BC. These PGs play a role in creating a pro-tumourigenic ECM through adjusting mechanical properties and biochemical signals. PGs and the ECM, however, interact dynamically, highlighting the complexity of the TME and thus that targeting PG-mediated ECM remodelling may be a potential strategy in BC therapies.

PGs are a large family of proteins that are tightly glycosylated and contain linear GAG chains [5]. So far, an estimated 43 PGs have been characterized, and classifying them has been very convoluted, because it is not only the protein part that varies but also the glucydic moiety [6]. Indeed, the sequences of glycans chains differ significantly due to their activity not being governed by the genetic code but by enzymatic activity and are also modified by the addition of sulfate and uronic acid groups [7].

**Figure 1:**
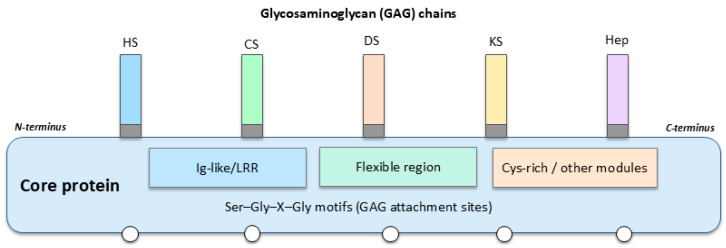
General structure of a PG and determinants of functional diversity.

As illustrated in Figure 1, a generic PG consists of a modular core protein bearing multiple Ser–Gly–X–Gly motifs to which GAG chains are covalently attached. Different combinations of core protein domains (for example, Ig-like, leucine-rich repeat, and cysteine-rich modules), together with the number, type, and position of GAG chains (HS, CS, DS, KS, or Hep) and their sulfation patterns, generate structurally distinct PG species. These structural variables, in combination with subcellular localization and the mode of membrane association (intracellular, cell-surface, pericellular/BM, or secreted ECM PGs), underlie the functional differences in ligand binding, ECM integration, and cell signalling that are particularly relevant in BC.

GAGs can be of five types: heparin (Hep), HS, CS, KS, and DS (Figure 1). All are sulfated and hence, highly negatively charged [8]. Despite their great diversity, PGs are highly conserved among life beings and are commonly found in the ECM, forming complexes with several proteins such as collagens or free GAGs such as hyaluronan (HA) and so on [9]. Crucially, although HA is often observed in association with PGs in large ECM aggregates, it is not covalently connected with PGs’ core proteins. Rather, HA binds non-covalently to specific PGs—especially versican—via specialized link modules that facilitate the formation of voluminous pericellular complexes. These assemblies of HA-PGs control fundamental cell processes, as they govern key cellular processes such as adhesion, migration, and signalling that influence BC progression. In addition, PGs, as glycoproteins, constitute basic building blocks of glycocalyx. Changes in this layer are well characterized in an array of diseases, impairing immune and vascular functions and, in cancer, contributing to progression and metastasis [10,11,12,13,14]. In such an environment, PGs have notable significance in oncology, and it is crucial they not be overlooked.

**Figure 2:**
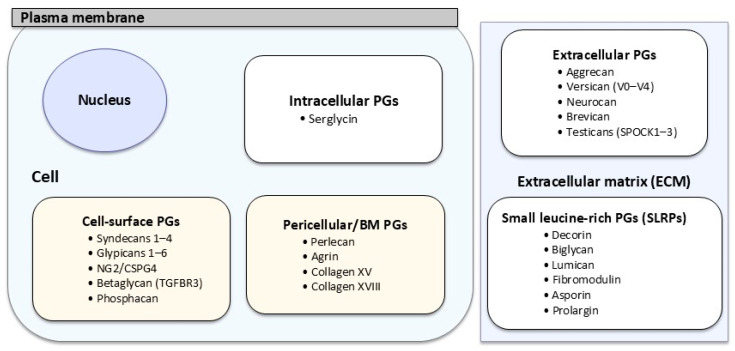
Schematic representation of PG families according to subcellular localization in BC.

However, this classification has not been as rigorous as suggested by the GAG chain, and the proposal made by R. V. Iozzo et al. is much more accepted nowadays [15]. In short, these authors used cellular location, gene/protein homology, and the presence of specific protein modules. Thus, PGs were categorized into four major groups (Table 1). The first group consists of intracellular PGs with only one example. The second group comprises those PGs located on the cell membrane and contains two further subgroups, as these are transmembrane proteins or glycosylphosphatidylinositol (GPI)-anchored proteins. The third group identifies a small set of PGs localized in the surroundings of cells that are part of the BM. Lastly, the fourth group is the largest and is classified in three hierarchical subgroups named hyalectan-lectican PGs, a spock PG, and small leucine-rich proteoglycans (SLRPs). The latter can be further subdivided into canonical SLRPS (three classes) and non-canonical SLRPS (two classes) (Figure 2).

Given the growing evidence of PGs as key regulators of BC progression, this review aims to synthesize existing knowledge by mapping their structural and functional diversity across a classification-based construct. The sections systematically target PG subfamilies (intracellular, cell-surface, pericellular, extracellular, and small leucine-rich), as well as non-classical members, to elucidate their context-bound roles in tumour cell behaviour, microenvironmental remodelling, and intercellular communication. We provide this integrated view of PG distribution, which refines the conceptual landscape of BC biology and adds relevance to PGs as diagnostic and prognostic biomarkers, liquid biopsy markers, and emerging therapeutic targets. Understanding PGs has therefore become a pivotal part of BC biology workflows.

Given the increasing recognition that individual PGs exert highly specific and sometimes opposing biological effects on BC progression, Table 2 provides a concise and integrated functional overview summarizing those PGs for which direct roles in tumourigenesis, invasion, or metastatic dissemination have been reported. Complementing the structural classification presented in Table 1, this table brings together experimental evidence across PG families and specifies whether each molecule predominantly promotes or suppresses primary tumour growth, modulates invasive and migratory behaviour, or contributes to the establishment of metastatic niches. By mapping these functional consequences, Table 2 helps contextualize the mechanistic sections that follow and clarifies the distinct and subtype-dependent contributions of individual PGs to BC biology.

Since the structural diversity and functional specificity of PGs are largely determined by the composition and sulfation patterns of their GAG chains, Table 3 presents a comparative overview summarizing the principal GAG types and characteristic sulfation features associated with the major PG families discussed in this review. These post-translational modifications (PTMs) critically influence ligand binding, signalling specificity, ECM interactions, and downstream communication, thereby shaping the distinct biological roles of individual PGs and ultimately contributing to BC progression. Including this synthesis at this point in the manuscript offers a structural framework that supports and contextualizes the mechanistic analyses developed in the subsequent sections.

## 2. Intracellular PGs

Serglycin is very special, because serglycin is the only member of the intracellular PGs group. The lone PG harbours Hep chains (though alternative GAGs may be attached depending on the cell type), but in BC cells, serglycin is generally loaded with CS chains [16]. On its function, it was primarily studied in mastocytes, in which it could be the channel through which proteins are delivered into granules and thereby the secretion rate of protein to the extracellular medium [17,18]. However, in BCs, enhanced expression levels were shown to be associated with more malignancy of the cells by promoting epithelial–mesenchymal transition (EMT), chemoresistance, and enhanced expression of matrix metalloproteinases (MMPs) like MMP-1, -2, -9, and -14 together, as well as, most importantly, anchorage-independent growth and enhanced extravasation and metastasis [16,19,20,21,22,23,24]. However, even though the role of serglycin as a reservoir has yet to be better studied, it was determined that the overexpression of serglycin to exert the shown effects was achieved only through CS chains, because the deglycosylated form did not stimulate all these cellular functions completely [16]. In fact, alterations in the balance of glycoenzymes involved in the synthesis of these chains also regulated cellular behaviour similarly [25].

## 3. Cell-Surface PGs

The cell-surface PGs are composed of 13 variants. Out of them, seven syndecans, neural/glial antigen 2 (NG2), betaglycan, and phosphacan are transmembrane proteins, while the six glypicans are GPI-linked PGs (Table 1). The majority of such cell-surface PGs contain HS side chains, except for NG2 and phosphacan, which contain CS, and betaglycan, which carries both HS and CS oligosaccharide chains. These HS complexes bind to ligands, cytokines, chemokines, and growth factors, as they serve as a reservoir and act as a gradient [270]. In addition, the PG extracellular domain may be dislodged, and soluble HS fragments can be produced with further biological impacts [271].

### 3.1. Syndecans

Syndecans are a family of four single-pass transmembrane PGs that comprise an intracellular domain, a transmembrane domain, and an ectodomain that, upon disordering, increases the diversity of the biological processes in which they function [272]. This ectodomain may also involve the consensus sequences in which HS oligosaccharide chains are linked, though other GAGs could also be present. Higher levels of syndecan-1 are associated with greater tumour size, enhanced malignancy, promotion of angiogenesis, and greater risk of metastasis [26,27,28,29,30].

The MMP-mediated cleavage of the C-terminal fragment of syndecan-1 has also recently been implicated in the development of this aggressive phenotype seen in BC, and its inhibition serves to reduce cell proliferation [31]. Moreover, lower expression of syndecan-1 in the triple negative SUM-149 cell line shows concomitantly reduced expression of interleukin-6 (IL-6), interleukin-8 (IL-8), and epithelial growth factor receptor (EGFR) with definitive effects for the formation of the aggressive inflammatory BC phenotype [32]. Furthermore, there is an inverse correlation between ER and syndecan-1 expression; the loss of ER results in overactivation of syndecan-1 and its effects, and, on the other hand, restoration of the ER signalling pathway constricts expression of syndecan-1 [33,34]. This close relationship between the two species is of interest. It points to more effective hormone therapies being developed, especially in cases of resistance.

Recent findings have expanded the definition of syndecan-1 beyond its canonical functional roles in proliferation and adhesion. A recent study showed that syndecan-1 over-expression induces a procoagulant phenotype in BC cells via upregulation of the tissue factor (TF) pathway, leading to increased thrombin generation and platelet activation [35]. Such a result links syndecan-1 to hematogenous dissemination, thus integrating its angiogenic and metastatic function in the surrounding TME at large. These findings were complementary and identified syndecan-1 as one of the genes in a five-gene proteoglycan signature closely linked with BC aggressiveness, which lend support to its potential clinical utility as a prognostic marker [36]. Furthermore, syndecan-1 was reported to regulate metabolic reprogramming and glucose metabolism, extending its role to not only ECM modulation, but also the metabolic adaptation of tumour cells [37]. Together, these reports characterize syndecan-1 as a multi-directional influence modulator, controlling coagulation, metabolism, and intercellular signalling in BC progression.

Regarding syndecan-2, it is also upregulated in BC and promotes the invasion and migration of cancer cells by reorganizing the actin cytoskeleton [38]. On the other hand, downregulating syndecan-2 induces apoptosis and decreases metastasis [39,40]. Similarly to syndecan-1, the expression of syndecan-2 is dependent on the ER status and on insulin-like growth factor receptor (IGFR) and EGFR; as well, it affects the malignant behaviour of BC cells [41].

The mechanistic explanation indicated that syndecan-2 activates the Wnt/β-catenin pathway, inducing EMT and cell invasion [42]. This pathway contrasts with co-receptor and proteolytic roles noted for syndecan-1 and illustrates the functional heterogeneity in the syndecan family members. Although there are limited reported findings on syndecan-3 in BC, existing evidence has suggested that syndecan-3 is linked to hypoxia-associated and glycolytic gene signatures and interacts with the tissue factor pathway inhibitor (TFPI), affecting tumour-associated coagulation [43,44,45].

Recently, a large expression of syndecan-3 mRNA in early-stage breast carcinomas were found, which was strongly correlated with hypoxia-responsive and metabolic gene modules, especially Notch, Wnt, and Hedgehog (Hh) pathways [46].

On the first analysis, syndecan-4 was not overexpressed in BC. Nonetheless, additional studies showed that the increased expression of syndecan-4 is associated with a better prognosis in BC patients, except, notably in ER-subtypes, it was demonstrated that this isoform is also under hormonal regulation [47]. Its silencing, interestingly, induced enhanced cell adhesion to the ECM and decreased invasive properties [47], complicating the involvement of syndecan-4 in BC. In fact, they would also support the importance of a GAG moiety with HS chains interacting with other protein factors such as autotaxin (ATX), LL-37, and Wnt signalling factors, leading to the effect on cell behaviour [48,49,50].

In TNBC cells, syndecan-4 was also shown to promote angiogenesis and VM through the modulation of VEGF and IGFBP-1 secretion and cytoskeletal organizational mechanisms. The silencing of syndecan-4 impaired these processes and reduced the invasive potential of tumour cells, highlighting its contribution to the formation of alternative vascular networks that sustain tumour growth.

Recent works have also clarified the function of syndecan-4 in BC. Syndecan-4 was also demonstrated to promote angiogenesis and VM in TNBC cells through the regulation of VEGF and IGFBP-1 secretion and cytoskeletal organizational mechanisms. The silencing of syndecan-4 impeded these processes and diminished the invasive potential of tumour cells, underscoring its role in shaping the formation of alternative vascular networks supporting tumour growth [51].

Taken together, recent studies indicate that syndecan function in BC acquires a more diverse face. Syndecan-1 modulates coagulation and metabolic adaptation; syndecan-2 favours EMT and invasion via the activation of Wnt/β-catenin; syndecan-3 initiates early metabolic adaptation; and syndecan-4 acts on hormones contextually, regulating the cytoskeleton, angiogenesis, and VM in TNBC. Syndecans are also dynamic modulators that serve to coordinate cell signalling (and, conversely, metabolism) and interaction with the microenvironment in an integrated view. Thus, the potential target for therapeutic intervention should be specific syndecan isoforms and pathways themselves rather than generalized to all family members.

### 3.2. Neural/Glial Antigen 2 (NG2)

NG2, or the neural/glial antigen 2, is emerging as an important PG for understanding BC and other neoplasms because of its strong association with tumour progression and metastasis [52]. Its overexpression has been correlated with poor clinical outcomes and diminished therapeutic responses in BC, particularly in the triple-negative breast cancer (TNBC)/basal-like subtype, which remains one of the most aggressive and difficult to treat [53].

With its expression closely linked to pericytes, NG2 is thought to play a fundamental role in regulating tumour microvasculature and, consequently, in mediating the extravasation of tumour cells [54,55,56]. Moreover, since NG2 is also expressed in tumour cells of the mesenchymal phenotype, adipocytes, and cancer-associated fibroblasts (CAFs), its contribution to tumour progression appears to be even broader and more complex [57,58].

Given this multifunctional involvement, NG2 has been proposed as a promising target for chimeric antigen receptor (CAR)-T-cell therapy in several malignancies, marking a significant advancement beyond the early efforts to develop immunotherapeutic approaches for TNBC and basal-like cancers [59]. Likewise, alternative therapeutic strategies aimed at eliminating NG2-positive cells are currently being explored [60,61,62], and this research direction has even been extended to other tumour types such as melanoma [63,64].

At the molecular level, NG2 functions as a cell-surface PG that interacts with multiple extracellular ligands to regulate adhesion and survival signalling. For example, an interaction has been observed between NG2 expressed by tumour cells and collagen VI (COL-VI) expressed by mammary stromal adipocytes that results in growth-stimulatory and pro-survival effects in BC cells [64]. Indeed, the mere presence of COL-VI can facilitate tumour invasion through the mammary stroma, underscoring the crucial role of NG2 in BC progression and highlighting the broader importance of understanding the composition of the matrisome.

Furthermore, NG2 contributes to the establishment of a supportive bone marrow metastatic niche and the induction of tumour cell quiescence, a dormant state that can be maintained when NG2 function remains unaltered by ageing or environmental influences [65].

A wealth of evidence that supports the significance of NG2 as a critical glycoprotein involved in the pathophysiology of BC has yielded results. Notably, one study showed that antibody–drug conjugates specifically targeting NG2 displayed significant cytotoxic activity in TNBC cell lines and further supported its role in the development of a clinically relevant therapeutic target [66].

### 3.3. Betaglycan

Betaglycan, also known as TGFBR3 or transforming growth factor beta receptor III, is a HS/CS cell-surface PG that binds transforming growth factor (TGF) ligands through its core protein and fibroblast growth factors (FGFs) via its HS chains. However, rather than acting as a canonical receptor or transducer, betaglycan primarily regulates ligand availability and modulates TGF–receptor interactions, thereby influencing cellular behaviour through indirect mechanisms [67,68].

In BC, TGFBR3 plays a complex and context-dependent role. Its functional outcome is influenced by whether it remains anchored to the membrane (TGFBR3) or is released into the extracellular space as a soluble form (sTGFBR3). Both isoforms differentially modulate TGF-β activity and, consequently, cell fate. An overall loss of TGFBR3/sTGFBR3 enhances the TGFβ signalling pathway, thus favouring progression. This observation, however, highlights the complexity of its function, as TGFBR3 has been identified as having both an anti- and a pro-tumour role [69,70,71].

Regarding the soluble form, sTGFBR3 can sequester TGF ligands, reducing their pro-tumoural effects; however, clinical data remains inconsistent. Some patients show elevated plasma sTGFBR3 levels associated with decreased tissue sTGFBR3 expression, complicating its potential use as a reliable prognostic marker [72,73,74]. Mechanistically, betaglycan has been linked to Cdc42, a small GTPase regulating cell morphology, adhesion, and migration, where imbalance may shift cell fate toward invasion [75,76,77]. Furthermore, betaglycan mediates α5β1 integrin trafficking, enhancing cell adhesion and supporting epithelial integrity, which aligns with its basolateral localization in healthy tissue [78]. This role of TGFBR3 in supporting tissue integrity is also reinforced by its involvement in cell polarity, given that, in healthy tissue, it is located basolaterally [79]. Additionally, TGFBR3 appears to influence the activity of CAFs, further shaping tumour–stroma interactions [80].

Recent BC studies have confirmed that TGFBR3 downregulation is directly involved in advancing cancer by increasing TGF-β/SMAD signalling and promoting EMT [81]. Re-expression of TGFBR3 in BC models in experimental study results reduced invasiveness, re-established epithelial polarity, and partially reversed mesenchymal traits, indicating its function as a suppressor of metastatic dissemination of BC. Further, clinical studies demonstrate that reduced levels of TGFBR3 is associated with an advanced stage in tumour cancer, a poor overall survival phenotype, and, in particular, with hormone receptor-negative and triple-negative tumours. Together, these observations place betaglycan as an essential glycoprotein modulator of TGF-β signalling and a potential prognostic biomarker in BC.

### 3.4. Phosphacan

Phosphacan is another single-pass transmembrane PG, and it interacts with the neural cell adhesion molecule (NCAM), tenascin, or contactin [82]. Therefore, it plays a role in cell adhesion like other cell-surface PGs. In addition to these ligands, a long list of others has been described: pleiotrophin (PTN), interleukin-34 (IL-34), midkine, β-catenin, vascular endothelial growth factor (VEGF), hypoxia-inducible factor (HIF)-2, or ErbB4 [83]. Of these, the first two are the best studied in BC, although there are still many unresolved questions. Regarding the phosphacan/PTN interaction, an overactivation of this signalling pathway has been observed precisely due to an overexpression of PTN in several BC subtypes. However, it is in the TNBC/basal-like subtype where this relationship is most evident, and in fact, this molecular feature reveals a greater aggressiveness of the tumour. This aggressiveness is related to anaplastic lymphoma kinase (ALK)-mediated overactivation, an important oncopromoter [84,85]. Alternatively, the phosphacan/PTN axis has also been linked to an overactivation of cyclin-dependent kinase inhibitor 1A (CDKN1A), a protein involved in cell-cycle regulation that is particularly affected during chemotherapeutic treatments and induces chemoresistance [86,87]. On the other hand, IL-34 could be behind the better prognosis of BC luminal and positive human epidermal growth factor receptor 2 (HER2+) tumours [88].

### 3.5. Glypicans

Glypicans are a family of six GPI-anchored PGs harbouring HS moieties with the capacity to influence cellular morphology, thus the differentiation. In fact, most of them are essential for embryonic development. Among them, glypicans 1, 3, 4, and 6 have been found to be involved in BC. Others, such as glypican-2, with no links to BC to date, are involved in lung cancer or childhood tumours, while glypican-3 is more generically affected in several types of cancer [89]. It is a family of proteins that has gained prominence in recent years and are potential therapeutic targets. These data may suggest that glypicans do not have reiterative functions, and this has been demonstrated, for example, by their prognostic role in BC. While overexpression of glypican-3 correlates with a better outcome, it is the low levels of glypicans 1 and 6 that have been associated with longer survival rates [90]. However, it depends on the BC subtype [90]. Furthermore, given its role in cell development and differentiation, it is not surprising that its expression is strongly influenced by age, as has been found with glypican-1, and its role in regulating the proliferative effects of fibroblast growth factor 2 (FGF2) in breast skin, as its expression largely decreases in older women despite being the major glypican in this tissue [91]. In addition, glypican-1 participates in multiple signalling pathways regulated by other FGFs, VEGF, TGF-β, Hh, or the bone morphogenic protein (BMP) [92]. This broad influence of glypican-1 in cell signalling may explain why its expression has been found to increase significantly during BC progression, but it has not been possible to trace a clear relationship with changes in other biomarkers [93].

Recently, in BC cell lines, it has been suggested that EGFR and JAK/STAT pathways influence the expression of multiple glypicans (including glypican-1 and glypican-4), indicating that extracellular signal-regulated networks might regulate glypican expression and hence tumour behaviour [94].

Interestingly, we need to recall again the existence of interactions between glypican-1 and COL-V, a minor fibrillar collagen that regulates pore size in the interstitial matrix, as a recent study has shown that loss of COL-V expression may be associated with problems in the activity of glypican-1 as a regulator of FGF2 [95]. Therefore, COL-V would be a glypican-1-mediated tumour suppressor, although it has already been indicated that the role of this collagen may also be associated with pro-tumour effects, too. Therefore, understanding these cascades of interactions between matrisome components would help to better define the processes of tumour progression.

Another glypican whose low expression levels have been associated with a better outcome is glypican-6. In this case, a correlation has been found between glypican-6 and the nuclear factor of activated T cells (NFAT), a transcription factor that induces the activation of several pro-oncogenic and pro-migratory genes and which would cause the dysregulation of the Wnt/β-catenin signalling pathways through the mediation of glypican-6 [96]. In contrast, glypican-3 inhibits the advance of BC and metastasis precisely by affecting the Wnt/β-catenin signalling pathways, too [97], which is rather challenging, as the activation of the non-canonical Wnt pathway concomitantly to the de-activation of the canonical Wnt pathway was also found in glypican-3. Precisely, in this work, the authors identified that glypican-3 was released into the extracellular milieu to compete or interact with Wnt and reduce cell invasiveness [97]. However, it is not yet entirely clear how it might differ from glypican-6 for the final effects to be so opposite. Alternative approaches have also identified that glypican-3 modulates the activity of the p38MAPK signalling pathway to the extent of inducing an EMT that would reverse the tumour cell phenotype and induce a reduced metastatic potential [98,99].

In recent years, glypican-4 has been described for the first time as having a tumour-suppressive potential, in the sense that downregulation would favour progression of the BC [100]. Hence, glypican-4 would probably be classed as glypican-3. In addition, it was also reported that plasma levels of circulating glypican-4 in patients with ER^+^/HER2^−^ metastatic BC predicted 24-month overall survival, indicating the potential of glypicans not only as cell-surface modulators but also as non-invasive prognostic biomarkers in BC [101]. So glypican-1 and glypican-6 serve largely as tumour promoters, and glypican-3, and glypican-4 inhibit BC. Their control by the EGFR and JAK/STAT pathways suggests their central place in the tumour signalling pathways. Therefore, glypicans are proposed as biomarkers with therapeutic targets in BC.

## 4. Pericellular PGs

The pericellular PGs group comprises four protein species (Table 1). Although they are found surrounding the cell membrane, they differ from cell-surface PGs in that they are not anchored to the membrane but establish non-covalent interactions with integrins, cluster of differentiation 44 (CD44), or other membrane proteins [15,273]. They can also be considered part of the BM, although a difference with the extracellular PGs is that this group is contained in the pericellular matrix (PCM). It is a spatial construct initially conceived for chondrocytes, thusly called chondron [274]. This PCM acts as a transducer of both mechanical and biochemical signals between the cell and the external ECM. Therefore, various extracellular PGs and fibril and non-fibril-forming supporting collagens can also be found in the PCM, accepting it is a diffuse delimited boundary. Because of this composition, PCM PGs play a key role in the storage and diffusion of a myriad of extracellular regulatory proteins such as the growth factor, morphogens, enzymes, and all other matrisome proteins [275]. However, unlike intracellular, cell-surface, and extracellular PGs, the PGs that fall into this group are not yet well understood in terms of their participation in BC, at least except for both multiplexins (COL-XV, -XVIII).

### 4.1. Perlecan

Among other functions, perlecan regulates angiogenesis and lipid metabolism, mainly by modulating the functions of FGF2, VEGF, the platelet-derived growth factor (PDGF), and other factors acting as a mediator of the TGFβ signalling pathway [102,103,104]. Interestingly, perlecan plays both pro- and anti-angiogenic roles via the N-terminal HS glycan moieties or the C-terminal fragment named endorepellin, respectively, thus promoting or inhibiting cell migration and extravasation [105,106]. Therefore, the expression levels of perlecan, as well as the extension of its modifications by matrix-degrading enzymes such as MMPs, heparanases, or sulfatases, may have a determinant role in the tumoural cell fate. Briefly, several analyses show a complex situation in the expression and distribution of perlecan in breast tissue. While in healthy tissue it is restricted to epithelial and vascular tissues, in neoplasms, the epithelial expression of perlecan almost disappears, showing localized foci and a substantial increase in the stroma, which, in addition, coincides with an increase in plasma levels of this PG [107,108].

In addition, perlecan as HS-containing PG controls the gradient of absorbed ligands, and it was already demonstrated that these gradients govern breast tumoural cell behaviour in hydrogel matrixes, which may be associated with perlecan-enhanced stroma in tumoural lesions [109]. In view of all these data, recent speculation has been that perlecan may be involved in the development of BC and other cancers [110]. Its localized expression, initially, tends to serve as a damper on tumour growth, but the action of various enzymes released into the ECM and perlecan induces molecular switching of the pericellular environment, thus stimulating tumour spreading and metastasis [111]. Due to the midpoint of the tumour between a local and higher capacity for spreading, perlecan has been suggested as a target for therapy; many of attempts are currently being re-specifically demonstrated in BC [111]. In a recent immunohistochemical study of invasive female breast carcinoma, higher perlecan (HSPG2) expression was positively correlated with aggressive features (higher grade of tumour, advanced T stage and lymphovascular invasion, and high Ki-67 index and HER2 positivity), thereby confirming its use as a marker of BC progression [112]. In BC, perlecan upregulation is indicative of a higher grade of tumour and HER2+ as a whole. Its transition from epithelial to stromal localization is a functional transition favourable to metastasis. Therefore, targeting perlecan or its enzymatic modifiers may potentially fill in for novel therapeutic applications in aggressive BC subtypes.

### 4.2. Agrin

In contrast to the situation described for perlecan, most of the information available on agrin has been obtained in nervous tissue, and much less in other ones. In addition, it has been found to be particularly implicated in neuromuscular diseases [113,114], and there is also some overlap between the mechanisms of function of perlecan and agrin, both in their function of reserve and maintenance of gradients by the GAG fraction and in their capacity to release peptide fragments with biological activity [15,114]. Mechanistically, little is known about the role of agrin in BC evolution, but, ironically, it is a protein that is frequently overexpressed in BC in the proteomic analyses that have been performed [115,116]. Similarly, it also appears as a frequently mutated gene in genomic sequencing analyses [117]. And, interestingly, in both omics fields, it has shown a good ability to discriminate between early stages of Br and advanced BC with metastasis. This conclusion is not only valid in BC, but is also corroborated in other types of tumours, such as lung cancer [118]. Even so, it remains to be studied further to at least reach the level of knowledge we have with perlecan; although, thanks to these mentioned proteomic approaches, we can anticipate a role for agrin in BC linked to the angiogenesis and the alteration of the neuromuscular junctions. Suppression of agrin has been shown to boost CD8^+^ T-cell recruitment in BC models and block tumour progression, and a new trial determined that agrin in the BC model was acting as an important component of structural ECM, modulating the immune microenvironment, and may even be a new therapeutic opportunity in BC [119].

## 5. Extracellular PGs

Extracellular PGs comprise the largest group, and it is divided into three categories: hyalectan/lecticans, secreted protein acidic and rich in cysteine/Osteonectin CWCV and Kazal-like domain proteoglycans (SPOCKs), and SLRPS [15]. The latter is further subdivided into canonical SLRPS, comprising three classes, and non-canonical SLRPS, with just two additional classes [15]. These five classes of SLRPS have been defined by phylogenetic studies [120]. In total, 25 extracellular PGs have been described (Table 1).

### 5.1. Hyalectans/Lecticans

Hyalectans/lecticans are a group of four extracellular PGs named aggrecan, versican, neurocan, and brevican, with a shared structure in which the globular domain G1 N-terminal sequence interacts with HA, and the G3 C-terminal is an EGF-like/C-type lectin region [121]. The central domain contains several GAGs moieties, which are usually CS oligosaccharide chains [122]. Due to this protein structure, they tend to act as bridges between the cell surface and the ECM by interacting with HA and extracellular proteins. However, their histological expression is largely constrained, as aggrecan is mainly found in cartilage, and neurocan and brevican are almost exclusive in neural tissues [122,123,124,125]. However, there are some reports of the presence of alterations in the aggrecan gene expression as well as brevican methylation status in mammalian tumours [126,127,128].

With respect to aggrecan, the earliest work observed its overexpression along with other chondrocyte biomarkers in a rare BC variant named matrix-producing carcinoma of the breast [127]. More recently, however, it has also been found to be affected in the TNBC/basal-like subtype, so that overexpression of the *ACAN* gene is an early event that also identified those cases with a higher risk of disease recurrence [128]. For brevican, its hypermethylation is frequent in low-grade BC cases and correlated with an increased risk of metastasis [141]. Interestingly, in a cellular assay with a lineage of multidrug-resistant BC cells, it was observed that the presence of exogenous brevican in the culture medium interfered with the formation of HA-CD44 complexes, which sensitized the cells against doxorubicin [141]. This is an interesting line of work that has not been taken up again since this publication.

**Figure 3:**
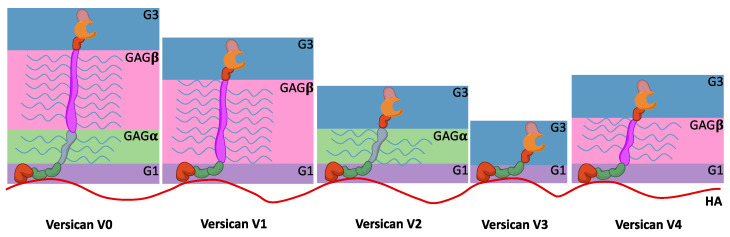
Domain structure of five splice variants of versican. G1 domain binds to the HA chain drawn in red, and all of them share the G3 domain. CS chains are shown in the form of blue lines. V0 shows both the GAGα and GAGβ domains, V1 just the GAGβ, and V2 the GAGα. V3 splice variant lacks both the GAGα and GAGβ domains, whereas the V4 shows a truncated GAGβ.

In contrast, versican, whose name comes from versatileness, has a broader tissue distribution. To date, five different isoforms generated because of splice variants have been described (Figure 3). The globular domains, G1 and G3, are present in all of them, similar to other hyalectans. G1 consists of an Ig-like module followed by two linker modules, while G3 contains the EGF-like repeat, the lectin-like module, and a complementary regulatory protein-like module (Figure 3). The two core domains, GAGα and the larger GAGβ, contain the CS chain molecules. In addition, these two core domains introduce the full range of versican splice variants. The full-length versican V0 contains the four aforementioned domains, versican V1 has the sequence G1-GAGβ-G3, and versican V2 shows the alternative sequence G1-GAGα-G3, while versican V3 lacks both GAGα and GAGβ [129,141]. Finally, there is also the versican variant V4, which shows a truncated GAGβ. This last isoform has been specifically identified in BC [130]. It facilitates cell adhesion and migration, establishing a temporary versican-enriched ECM that allows the interactions with proteins such as P-selectin, CD44, or Toll-like receptors (TLR) [131]. Therefore, this matrix mediates inflammation processes.

In addition, it also contributes to BC fate, as it was shown that it correlates with the presence of infiltrating pro-angiogenic tumour-associated macrophages (TAMs) in the peritumoural region of primary breast lesions as well as of lung metastatic tumours in canine and murine BC models [131]. Regarding BC cells, versican and the sulfate synthase PAPSS2 affect their migration rate and metastatic potential, as high versican levels or, alternatively, a high PAPSS2 activity correlated with an enhanced migration activity [132]. Although both CS chains and G1 and G3 domains have been associated with these cell effects [133], earlier studies about the role of versican in BC focused on the protein fraction [134,135,136,137,138]. More specifically, the G3 domain alone can induce tumour colonies, as well as increasing angiogenesis, cell proliferation, cell migration, metastasis, and chemoresistance.

Recent evidence reinforces the importance of versican in the TME of BC. Elevated expression of this PG in peritumoural mammary adipose tissue has been linked to enhanced proliferative activity, increased body mass index, and greater tumour aggressiveness, suggesting that versican mediates the metabolic–stromal interplay driving tumour progression [139]. Furthermore, its accumulation in the stroma and reduced proteolysis correlate with decreased CD8^+^ T-cell infiltration, supporting its potential as a biomarker for immune exclusion and therapeutic resistance [140].

### 5.2. SPOCKs

Secreted protein acidic and rich in cysteine (SPARC)/Osteonectin CWCV and Kazal-like domain PG, abbreviated to SPOCK, is a single-member subtype of extracellular PGs, also named testican, as it was first isolated in the seminal fluid. Later, the existence of the three testicans, testican-1, -2, and -3, has been identified. They share a protein sequence with five domains, one being the N-terminal domain I and the C-terminal domain V specific for this family, domain III is similar to the extracellular calcium-binding domain of SPARC proteins, and domain IV harbours the CWCV tetrapeptide sequence [15].

Interestingly, and despite their name, this set of PGs has been found to be significantly involved in various processes of neurogenesis, playing a role in cell-to-cell and cell-to-ECM interactions [142,143,144]. In other tissues, data are scarcer and little attention has been paid to them, although there is sufficient evidence to suggest that they influence the progression of several types of tumours, such as pancreatic, colorectal, gastric, prostate cancers, or gliomas [145,146,147,148,149]. Mechanistically, a link between testicans and MMPs is observed such that the enzyme activity and, consequently, its effects in the ECM and subsequent cell behaviour are influenced by the presence of fragments released from testicans by other MMPs [150,151,152,153]. It is therefore a complex regulatory cycle between metalloproteinases.

However, this is not the only involvement of testicans in ECM homeostasis. This is the case for finding testican-1 as an important mediator of acquired resistance to the HER2 targeted chemotherapy in gastric cancer [149]. In this assay, the chronic exposure of the SNU216 LR cell line to lapatinib induced resistance to this drug and many others, a more evident EMT phenotype, and the overexpression of testican-1, among other proteins. Intriguingly, after its knocking down by the addition of small interfering RNA, it was possible to restore the drug sensitivity.

Overall, these are interesting findings that should be considered in BC for several reasons. Firstly, because HER2 is also a frequently affected protein in this type of cancer, but also because acquired resistance to treatments occurs frequently in BC, if not the opposite, with patients classified as HER2+ that do not respond as expected to targeted therapy. More recently, an endothelial protein C receptor (EPCR)/SPOCK1 signalling axis has been discovered in BC [154]. EPCR has been shown to regulate the clotting process, inflammation, and apoptosis via activated protein C. It is also involved in many other biological functions, several of which are affected in cancer. However, EPCR also interacts with many other ligands, broadening the biological functions in which it participates, several of which are affected in cancer [155]. Indeed, the overexpression of EPCR in cancer cells has been associated with an increased anti-apoptotic response and metastatic activity [156]. The latter is where the EPCR/SPOCK1 signalling axis is located, as the expression levels of both proteins are directly correlated, as well as their activity, and the overexpression of SPOCK1 induces a higher potential for metastasis and colony formation [156]. Specifically, in luminal B and HER2+ subtypes, this link was much more evident [156].

Recent studies have expanded on this role even more. One study showed that SPOCK1 was significantly overexpressed in BC-associated fibroblasts and that silencing it decreased the fibroblast-mediated invasion and metastasis of breast tumour cells [156]. Another study showed that SPOCK1 was linked with stromal TGF-β signalling in TNBC, which drove EMT and poor prognosis [157]. Lastly, bioinformatic and immunohistochemical analysis determined that elevated SPOCK1 gene levels correlate with increased immune-suppressive macrophage infiltration and decreased CD8^+^ T-cell presence in high-risk BCs, suggesting a role in the immune remodelling of the tumour niche [158].

The combined data indicate that SPOCK1 in BC is not simply indicative of a tumour-intrinsic change and may be involved in a stromal–ECM–immune interaction. They have implications for targeting SPOCK1 or its pathways (TGF-β and MMPs) as combinatorial treatment in BC subtypes with high levels of stromal involvement.

### 5.3. SLRPS

The SLRP family is composed of 18 members; thus, they are the most abundant extracellular PGs (Table 1). As noted, this family is further subdivided into canonical SLRPS, with three classes named I, II, and III, and non-canonical SLRPS, with the two classes IV, and V. Of all the PGs, the SLRP family has the smallest average protein core size, with a solenoid leucine-rich repeat structure common for all the classes. Therefore, they present, in proportion, a higher abundance of GAGs chains, with the notable exception of non-canonical SLRPS, as they do not harbour GAGs oligosaccharide moieties, so they should not be considered true PGs [276]. Within the canonical SLRPS, class I PGs usually contain CS or DS, whereas class II PGs are rich in KS; class III, in sharp contrast, is the most heterogeneous, with PGs harbouring DS/CS chains, KS chains, or not being glycosylated at all. Another difference that discriminates between canonical and non-canonical SLRPS is that the former has two characteristic protein modules: cysteine-rich motifs at the N-terminus and “ear repeats” at the C-terminus inserted within the LRRs [149,150,151,152,153,154,155,156,157,158,276,277,278]. In terms of their biological function, SLRPS are among the main constituents of organ-enveloping membranes [279]. In the ECM, they participate in the assembly of collagens, protecting them from the enzymatic proteolytic activity and modulating the biological activity of growth factors and other ligands in the matrisome.

Despite this abundance of forms and their important roles in the maintenance of the ECM integrity, few SLRPS have been found affected in BC. Eight canonical SLRPS have been documented, most of them belonging to class I. In addition, the participation of non-canonical SLRPS remains even more obscure, with occasional but promising reports related to the participation of chondroadherin or podocan.

A recent large-scale genomic analysis identified a distinct PG gene signature in BC that includes multiple SLRPs, revealing that the dysregulated expression of certain SLRP genes correlates with a poor prognosis and increased metastatic potential [36]. This study highlights that, beyond individual SLRPs historically linked to breast tissue (e.g., decorin or lumican), the broader SLRP family may contribute to BC progression via coordinated changes in ECM remodelling. The findings suggest that SLRPs might serve not only as structural ECM components but also as active modulators of tumour–stromal interactions and as potential biomarkers or therapeutic targets.

#### 5.3.1. Biglycan

Biglycan, in addition to interacting with other ECM proteins like collagen (COL-I and -IV) or elastin, is strongly linked to inflammatory processes due to its ability to interact with TLR2 and TLR4, as well as with the co-receptor CD14 [159,160,161]. The activation of this pathway leads to increased production of cytokines and other mediators involved in inflammation, such as tumour necrosis factor (TNF)-α, IL-1β, and CCL/CXCL motif ligands, promoting the recruitment of macrophages, B cells, and T cells. Interestingly, regarding macrophages, this signalling pathway promotes the tumour-suppressive M1 subpopulation [162]. However, biglycan can also drive macrophage differentiation toward the pro-tumoural M2 phenotype via CD44, although this same pathway may promote autophagy [163,164]. The link between autophagy and cancer progression remains controversial, as autophagy can act either as a tumour-suppressive mechanism or as a pro-tumoural process that enhances cell survival and tumour growth [165,166]. Moreover, CD44 has been identified as a co-receptor in the TLR signalling pathway [167].

Additional roles of biglycan in cancer include the regulation of angiogenesis through the upregulation of VEGFA expression, accompanied by elevated HIF and reactive oxygen species levels in a TLR2/4-dependent manner [168,169,170]. These overlapping and sometimes opposing mechanisms indicate that biglycan may act as a fine modulator of tumour–stroma interactions rather than as a simple structural ECM component. Recent large-scale transcriptomic and proteomic analyses in BC have reinforced this notion, identifying biglycan as a key hypoxia- and ECM-associated gene. Elevated biglycan expression has been linked to poor outcomes in hypoxic tumours and to the reactivation of dormant BC cells via metabolic reprogramming, supporting its role as a driver of metastatic recurrence [171,172].

Therefore, the role of biglycan in BC is complicated to define and appears to depend strongly on its localisation and the cellular context. In early studies, the presence of biglycan in the ECM was associated with a reversal of breast tumour cells to a quiescent phenotype [173]. Conversely, stromal biglycan expression has been correlated with worse outcomes, as its knockdown reduced metastasis, impaired angiogenesis, increased CD8^+^ T-cell infiltration, and enhanced chemotherapy efficacy [174]. These apparently contradictory findings reflect the dual nature of biglycan’s activity in BC. Its overexpression in tumour endothelial cells may explain its frequent detection in the sera of BC patients, while silencing its expression in these cells impairs migration and alters tumour-vessel morphology [175].

Regarding fibroblasts, biglycan^high^ HER2+ fibroblasts were less tumourigenic than biglycan^low^ HER2+ fibroblasts, which were related with the enhanced immunogenicity of the tumour mediated by the upregulation of MHC class I molecules and the downregulation of TGFβ [176]. Moreover, exogenous biglycan was able to shift the differentiation of biglycan^low^ MHC class I^low^ HER2+ fibroblasts to overexpressing MHC class I fibroblasts [177]. However, the expression of biglycan by CAFs was associated with a lower presence of CD8^+^ T cells, thus creating an immunosuppressive tumoural environment in the TNBC/basal-like subtype [178]. For tumour cells, the expression of biglycan by BC stem cells was associated with a more aggressive migration and invasion of these cells, whereas the loss of biglycan led to a reduced metabolism and a decreased tumourigenic phenotype [178].

Altogether, these data indicate that biglycan acts as a molecular regulator with dual and context-dependent functions in BC: while its stromal and endothelial expression promotes angiogenesis, immune evasion, and metastatic relapse, its absence appears to restrain tumour progression. Understanding this balance could guide its future use as a biomarker of tumour dormancy and stromal activation or as a therapeutic target to disrupt the pro-metastatic niche.

#### 5.3.2. Decorin

Decorin is the most investigated extracellular SLRP in cancer and BC by far. It belongs to class I canonical SLRPS, and it is known for its important role in collagen fibrillogenesis and wound healing [179,180]. However, decorin covers an even more extensive range of biological functions. To name some of them, it is associated with angiogenesis [181], inflammation [182,183], or cell autophagy [184]. It is therefore considered the “guardian of ECM” integrity and, by extension, is a pivotal effector in the TME [185,186].

Recent evidence has clarified that decorin is not merely a structural ECM component but a dynamic regulator of the stromal compartment in breast tissue. The downregulation of decorin in mammary fibroblasts was shown to activate the IL-6/STAT3/AUF1 axis, converting normal fibroblasts into tumour-promoting CAFs. This process enhances epithelial–mesenchymal transition, stemness, and angiogenesis in adjacent tumour cells, illustrating how the loss of decorin supports a pro-tumoural microenvironment [187].

Structurally, decorin shares some common structural and behavioural features with other PGs, such as carrying side chains of GAGs (usually in the form of DS, although CS is also frequent) and binding to growth factors such as TGFβ, collagens, and other matrisome compounds [188]. In cancer research, decorin is shown to have a key oncosuppressive function, offering a promising future as a potential adjuvant drug [189]. This is due to its effects on the immune system, to the ability to regulate autophagy and mitophagy, and, interestingly, to act as a pan-receptor tyrosine kinase (RTK) inhibitor [190]. This is of particular importance in BC, because this inhibition would counteract the proliferative effects of an enriched expression of EGFRs, an inhibition that is achieved by inducing a p21-mediated cell-cycle arrest and the upregulation of apoptosis [191].

More recently, decorin has been implicated in the regulation of tumour lymphangiogenesis. Experimental models demonstrated that recombinant decorin binds VEGFR3 on lymphatic endothelial cells, blocking VEGF-C signalling, reducing lymphatic vessel density, and limiting metastatic spread in BC xenografts [191]. These findings expand decorin’s anti-angiogenic function to the lymphatic system and reinforce its dual role in vascular suppression.

Of note, HER2 expression is a common biomarker for classifying the BC subtype and deciding the therapeutic schema. However, few studies have addressed the relationship between decorin and BC via EGFRs, with some evidence that would need to be conclusively proven [192]. Notwithstanding, decorin has been shown to be an important tumour suppressor in BC [193]. Regarding mitophagy in BC, decorin was able to induce it via mitostain and the peroxisome proliferator-activated receptor γ coactivator-1α (PGC-1α) [194]. Likewise, autophagy is also present, and it is induced by the Peg3/Beclin-1 axis, with Peg3 being a potent inductor for the expression of thrombospondin 1 (TSP-1), therefore linking this event with an increased angiogenesis [195].

Due to these strong functions in cancer behaviour, it is not surprising that there has been an attempt to trace the dynamics of expression of decorin in healthy and tumoural mammary samples. These studies clearly show that decorin is expressed by stromal cells in both healthy and cancer samples, and cancer cells do not express any decorin [196]. Paradoxically, plasma decorin levels increase in more advanced stages, even to the point of proposing decorin as a potential biomarker to identify patients in advanced stages [197]. However, it would have to be carefully analyzed as to whether these levels are due to the dysregulation and alteration of the cells surrounding the tumour lesion rather than coming from the cancer cells themselves.

In agreement with this, a recent PG gene-signature study in BC confirmed that high stromal decorin expression correlates with improved survival and reduced metastatic potential, reinforcing its prognostic value as a stromal-derived tumour suppressor [198]. Collectively, these data highlight that decorin functions as a central regulator of the BC microenvironment: its loss promotes fibroblast activation and tumour invasion, while its expression suppresses angiogenesis and lymphangiogenesis, consolidating decorin as both a biomarker of stromal homeostasis and a promising therapeutic target.

#### 5.3.3. Asporin

Asporin, another class I canonical SLRP, shows a high homology to decorin, although it does not possess GAGs, and a similar tissular distribution [199]. Asporin inhibits the TGFβ/Smad signalling pathway, an important mechanism in chondrogenesis [200]. As TGFβ1 also plays a central role in cancer, it can be expected that asporin would be affected in these diseases too. Moreover, this regulatory loop between asporin and TGFβ can be extended toward other matrisome proteins such as COL-II, as its expression is positively correlated with TGFβ levels [201].

Regarding BC, asporin demonstrated a dual role with tumour-suppressor and pro-tumoural activities. Whether one predominates over the other depends strongly on how advanced the tumour lesion is, as in low-grade lesions and high asporin levels are associated with a better outcome, whereas in more advanced ones, prognosis is significantly worse [202]. Of note, high levels of asporin are found in tumoural lesions and are even greater in the invasive ductal carcinoma tumours than in ductal carcinoma in situ lesions [203]. Other gene markers related to invasiveness, EMT, cell motility, and ECM degradation appeared to be upregulated, too [204]. Likewise, a similar study found that asporin was significantly upregulated in lobular carcinomas than in healthy ducts (fold change of 23.3), whereas it was lightly upregulated, although significantly upregulated again when comparing lobular carcinoma with ductal carcinomas (fold change of 3.9) [205]. In addition, alternative markers again pointed to an alteration of the cell adhesion and ECM within these histological BC subtypes [206].

Unfortunately, none of these studies have delved into the molecular mechanisms in which asporin is involved; sometimes the authors have not even discussed its impact in BC. Nevertheless, there is some evidence that, in addition to tumour cells, fibroblasts also produce asporin and it is influenced by the BC subtype [208]. Importantly, it was found that asporin expression is inhibited by IL-1β, which is mainly secreted by TNBC/basal-like cells, and, in turn, this drop would stimulate the TGFβ/Smad signalling pathway and the subsequent pro-metastatic and pro-EMT events, with an impact on the patient outcome [206].

More recently, experimental results have revealed interesting new insight into the role of asporin in the breast TME. Adipocytes were identified as an unexpected source of asporin in obesity-associated BC, which accumulates in mammary adipose tissue and enhances tumour progression. The genetic knockout of asporin markedly reduced tumour growth and improved CD8^+^ T-cell infiltration, thus highlighting that it mediates a stromal–adipose axis promoting tumour immune evasion and progression in obesity-linked BC [207].

In conclusion, asporin also serves as a context-dependent regulator of BC progression via the TGFβ/Smad pathway and demonstrates an enhanced sensitivity to the tumour subtype and microenvironmental stimuli. Its modulation by metabolic and inflammatory factors, and even more so in obesity-associated settings, also underscores the position of this factor as a mediator of a pathway between ECM remodelling and tumour immune evasion.

#### 5.3.4. Fibromodulin

Fibromodulin belongs to class II canonical SLRPS; therefore, it is expected to possess KS side chains and be highly expressed in the connective tissue bound to ECM compounds [207]. Specifically, fibromodullin binds to the major fibril constituents COL-I and -II to stabilize this fibrillar scaffold [208]. In addition, it also binds other compounds such as TGFβ1, FGF, VEGF, C1q, and other cytokines [209,210,211]. Regarding its role in cancer, it has been found to be a promoter of angiogenesis [212].

In BC, a regulatory loop has been described between fibromodulin and the Wnt/β-catenin, NF-κB, and TGFβ signalling pathways [213,214]. Overactivation of these signalling pathways is associated with enhanced proliferation, cell migration, metastasis, EMT, and a more aggressive phenotype. Briefly, fibromodulin levels appear to be upregulated as consequence of the activation of the Wnt/β-catenin signalling pathway [214]. From this finding, fibromodulin could be expected to participate as a further promoter in BC tumourigenicity and malignancy. Nevertheless, the overexpression of this PG inhibits the levels and subsequent effects triggered by TGFβ, as well as the NF-κB pathway, although the latter was found to be cell-line dependent [215].

Recent proteomic profiling of BC tissues shows that fibromodulin is one of the matrisome compounds enriched in metastatic lesions compared to primary tumours with associated ECM stiffening [216]. This emphasizes its pivotal structural and signalling role that facilitates invasive tumour phenotypes.

Fibromodulin, being a matrisome compound that is closely associated with collagens, is also an essential modulator of the interstitial fluid flux, stimulating a pressure gradient at the tumour/blood-tissue barrier and subsequently blocking the introduction of chemotherapy into the tumoural mass [218].

In addition, recent experimental findings indicate that the pharmacological inhibition of fibromodulin with RP4 as an antagonist efficiently inhibits Wnt/β-catenin signalling, leading to a decrease in migration and invasion seen in cancer models [217]. While not yet validated in BC, these findings support fibromodulin to be a potential therapeutic target to mitigate Wnt-driven tumour aggressiveness and metastasis. Dysregulation of fibromodulin is a common cancer event, yet its impact on tumour fate remains complex, exhibiting both tumour-suppressive and pro-tumour properties that vary according to the cellular and molecular environment.

#### 5.3.5. Lumican

Lumican is another class II canonical SLRPS, and its dysregulation has been widely described in various types of cancers, including BC, by affecting the inflammatory response, angiogenesis, and cell-to-ECM interactions [218]. Its expression has been known to be affected in BC since the 1990s [219,220]. However, the cellular and molecular mechanisms have not been described until more recently. Specifically, lumican has been shown to block cell adhesion via β1 integrins in melanoma [221], as well as promote neutrophil transmigration across endothelia via β2 integrins [222].

This link of lumican to integrins has also been found in BC. It interacts with α1, α2, α3, αVβ3, and αVβ5 integrins and promotes the overexpression of α2 and β1 integrins. In addition, downstream pathways linked to integrins and supporting cell proliferation, such as FAK and MAPK/ERK, appeared to be downregulated [223]. The final effect is that lumican induces cell-to-cell junctions and triggers the reprogramming of cell morphology toward the MET phenotype [224]. However, these effects are highly dependent on the cellular subtype, particularly on the ER status [223].

In addition to its regulation of integrins, lumican interferes with the formation of membrane protrusive structures that participate in cell motility. Whereas lamellipodia and filopodia appear in normal migrating cells, invadopodia and podosomes are actin-rich matrix-degrading structures closely associated with tumour invasion and metastasis. This degradative activity is mediated by cortactin and MMP-14, both of which are inhibited by lumican, resulting in the decreased stability of these membrane protrusions and the conversion of the invasive mesenchymal phenotype into a more epithelial one [225].

With respect to inflammation, lumican is associated with TLR4 and TGFβR1, which stimulate the innate immunity, macrophage recruitment and differentiation, and the TGFβ pathway [226,227].

In fact, a new multi-omics analysis has identified a positive relationship between the expression of lumican (LUM) and stromal scores, CAF infiltration, and poor responses to chemotherapy in BC, thus underscoring lumican expression as a stromal-derived regulator of TME and treatment resistance [228]. Generally, these findings imply that lumican functions as a structural ECM component and a regulatory molecule modulating tumour–stroma dynamics and thus the association of high stromal expression with poorer prognosis and therapeutic resistance in BC also.

#### 5.3.6. Prolargin

Prolargin, or proline/arginine-rich end leucine-rich repeat protein (PRELP), acts as a bridge between the BM and matrix collagens [229]. In addition, it is also involved in osteogenesis/osteolysis balance by inhibiting osteolysis [230]. As a consequence, and perhaps not surprisingly, prolargin was shown to prevent the development of bone metastasis in BC murine xenografts [231]. Indeed, the presence of prolargin in the primary tumour environment affected tumour growth by modifying tumour conditions [232]. Following these results, the authors proposed its use as a novel drug agent.

Interestingly, little progress has been made in this direction, since from then until two new papers were published in 2020, one on hepatocellular carcinoma and the other on bladder cancer, the role of prolargin has once again been highlighted [233,234]. In both types of cancer, when the expression of this PG was reduced, a higher risk of metastasis and a poorer outcome was observed. Such a reduction could already be observed even in the early stages, which reinforces the need for further research. In contrast, in pancreatic cancers, the presence of prolargin was associated with opposite prognostic outcomes [234,235].

Recent gene-expression profiling in BC identified PRELP among the PG genes whose decreased expression correlates with enhanced tumour aggressiveness and poorer survival, suggesting that PRELP acts as a stromal tumour-suppressive factor modulating ECM integrity and TGFβ-dependent signalling [36]. This study also highlighted that low PRELP levels were associated with reduced immune cell infiltration and increased EMT markers, reinforcing its importance in maintaining a suppressive TME.

In summary, evidence from both experimental models and gene-expression studies supports the protective role of prolargin in BC. Its ability to stabilize the ECM, limit bone metastasis, and modulate TGFβ and immune pathways places it as a promising biomarker and potential therapeutic target for controlling tumour progression and metastatic dissemination.

#### 5.3.7. Epiphycan

Epiphycan is a small DS/CS PG that participates in chondrogenesis, bone formation, and is abundant in the growth plate where it interacts with COL-IX [236,237]. Therefore, epiphycan has been associated with the scaffolding processes of the ECM.

In the context of cancer, its role, like that of many other PGs, remains poorly defined. Initially, it was observed that BC expression was significantly increased in response to the overexpression of Inhibin-β A, a member of the TGFβ family, by mesenchymal breast cells [238]. Intriguingly, the overexpression of Inhibin-β A had a direct impact on co-cultured breast epithelial cells by increasing their colony-formation potential [239]. Consequently, the Inhibin-β A/epiphycan axis revealed a reciprocal crosstalk between different cell populations involved in tumourigenesis, suggesting that its dysregulation could influence disease progression.

More recently, epiphycan alterations have been described in other cancer types. In prostate and ovarian cancers, epiphycan overexpression has been proposed as a marker of poor prognosis [240,241]. In ovarian cancer models, silencing EPYC significantly attenuated cell migration, invasion, and proliferation, indicating direct involvement in adhesion and signalling mechanisms [241].

In a recent integrative biomarker analysis, epiphycan was identified as one of the significantly overexpressed genes in malignant breast tissue compared to adjacent normal samples, with expression levels correlating with the tumour grade and reduced overall survival. These findings suggest that epiphycan may function as an ECM-driven promoter of breast cancer aggressiveness and could represent a potential diagnostic and prognostic biomarker [242].

Overall, these studies suggest that epiphycan plays a dual yet predominantly pro-tumoural role in BC, acting at the interface between stromal and epithelial compartments. By regulating ECM organization and cell-signalling pathways such as TGFβ, epiphycan may enhance tumour invasiveness and influence patient outcomes, reinforcing its potential as a stromal-associated biomarker and therapeutic target in advanced BC.

#### 5.3.8. Osteoglycin

Osteoglycin, as well as epiphycan, is another class III canonical SLRPS whose alteration in BC has been reported. Similarly, it participates in the assembly of collagens and in fibrillogenesis [243]. This structural role makes osteoglycin a relevant component of the ECM, contributing to tissue organization and stability.

Interestingly, osteoglycin, unlike other SLRPS, could be considered a tumour suppressor. Previous studies have shown that its overexpression in cancers such as colorectal cancer, hepatocarcinoma, gastric cancer, and BC improved prognosis [243,244,245,246,247,248]. Certain trends can be drawn from these studies with respect to their behaviour.

Firstly, osteoglycin induced EGFR dimerization and subsequent cell differentiation towards a more epithelial phenotype with reduced invasiveness. It also inhibits VEGF activity and enhances tumour-infiltrating lymphocytes (TILs) around the tumour. On the ECM side, gelatinase activity is reduced, ultimately decreasing the rate of tumour cell migration into adjacent tissues and lymph nodes. More specifically, in BC, osteoglycin represses the PI3K/Akt/mTOR signalling pathway.

In conclusion, osteoglycin appears to exert multiple tumour-suppressive effects in BC by simultaneously modulating EGFR, VEGF, and PI3K/Akt/mTOR pathways. Its dual regulation of epithelial differentiation and ECM remodelling supports its potential as a biomarker of favourable prognosis and a therapeutic target for restraining tumour invasiveness.

#### 5.3.9. Chondroadherin

Chondroadherin is mainly found in the extracellular space surrounding the chondrocytes located in the cartilage of the growth plate. Therefore, it is expected to be implicated in the proliferation processes and matrix assembly affecting this region. It mediates cell adhesion through α2β1 integrin and anchors to the ECM collagens COL-I, -II, and -VI, too [249,250]. In addition, chondroadherin competes with perlecan not only by interacting with the HS side chains via its C-terminal domain, but they both recognize the same ligand, the α2β1 integrin [251,252].

The chondroadherin/α2β1 integrin complex is involved in maintaining cell morphology and, consequently, influences motility, spreading, adhesion, and ECM fibre tension [252]. Unsurprisingly, the formation of chondroadherin/α2β1 integrin complexes by administering a chondroadherin peptide demonstrated a tumour-suppressor role in BC [253]. In addition, it reduced the risk of bone metastasis and was compatible with standard chemotherapy at low doses. Likewise, a lower expression of chondroadherin correlated with poorer outcomes in hepatocarcinoma, supporting the important role of chondroadherin in the regulation of cell morphology and adhesion [254].

However, chondroadherin may not behave the same way in all types of cancers. A later report found NFAT as an upstream regulator of chondroadherin in ovarian cancer, with increased expression of both proteins in later stages correlating with poorer outcomes [255]. Nevertheless, little is known about the molecular alterations behind this finding, and the function of chondroadherin remains elusive.

Although previous reports mainly highlighted the structural and adhesive roles of chondroadherin in the ECM, more recent findings indicate that its biological influence extends to intracellular signalling mechanisms relevant to tumour progression [256]. In BC, chondroadherin expression has been shown to decrease with increasing tumour malignancy, and its low levels correlate with poorer patient survival. The functional suppression of chondroadherin enhances cell proliferation and migration, while transcriptomic and biochemical analyses reveal the activation of the PI3K/Akt pathway under these conditions. These data support a tumour-suppressive role for chondroadherin, suggesting that it may inhibit breast tumour progression by stabilizing cell–matrix interactions and restraining oncogenic PI3K/Akt signalling activity. Collectively, these observations position chondroadherin as a potential prognostic biomarker and functional modulator of both extracellular and intracellular mechanisms in BC.

#### 5.3.10. Podocan

Podocan and podocan-like 1 are the only two members of the poorly investigated class V SLRPS. Initially identified in podocytes, the main cells of the epithelial visceral layer in Bowman’s capsule of the kidney, large amounts of these PGs have also been detected in the ECM of vascular tissue, suggesting expression by specific vascular cell types [257,258]. More specifically, podocan affects smooth muscle cells by reducing their migratory and proliferative capacities and increasing susceptibility to vascular injury [259].

Therefore, when translated to cancer, there is evidence linking podocan to the control of cell motility. For example, hypermethylation of *PODN* leads to lower mRNA expression and a worsened prognosis in gastric cancer patients [260]. Likewise, PODN gene expression decreases in osteosarcoma, with patients showing higher PODN expression having better survival rates than those with lower levels [261]. Interestingly, immune cell composition differs markedly between these subgroups, indicating a potential correlation between podocan and the tumour immune landscape [262]. Differences were observed in the number of Treg cells (higher in the low PODN group) and in mast cells (higher activated populations in the low PODN group) [262].

If similar differences exist in BC, they could help to explain the variability in tumour behaviour within molecular subtypes. Indeed, it has recently been observed that podocan expression levels are significantly decreased in BC patients, with therapy resistance and a shorter overall survival [263]. As for which cellular pathways are affected, the overexpression of podocan is associated with cell-cycle arrest at the G1/S transition by modulating p21 and cdk2 expression [263]. Furthermore, bioinformatic analyses suggest that collagens such as COL-VI, SMA, fibroblast activation protein alpha (FAP), and olfactomedin-like protein 2B may also be implicated in podocan activity [264].

More recent evidence in breast carcinoma models confirms that PODN is significantly downregulated in tumour tissue compared with normal breast tissue and that this reduction is associated with a more malignant phenotype in BC [36]. Specifically, the PG gene signature study identifies PODN among the downregulated genes in breast carcinomas and links its lower expression to advanced tumour stages and poor prognosis profiles. These findings support the hypothesis that podocan may act as a tumour suppressor in the BC microenvironment by maintaining ECM integrity and restraining tumour progression.

## 6. Non-Classified PGs

Before closing this section, we need to mention a group of part-time PGs mentioned in the work by R. V. Iozzo et al., whose role as a PG is not entirely clear (Table 1) [15]. For instance, we discovered proteins that remain of uncertain action in the field of oncology (leprecan), along with those of high interest, such as endocan (ESM1) and CD44, and proteins that can be attributed to other families (collagen types IX and XII (FACIT subgroup) [264,280,281,282].

In BC, endocan (ESM1), an endothelial-secreted PG containing GAG chains, has recently been identified as a promising candidate in blood to predict tumour burden and recurrence. Recently, a clinical translational study confirmed the correlation between circulating endocan levels and tumour load in both preclinical and patient models and detected the recurrence in the post-operative phase, even if existing markers such as carcinoembryonic antigen (CEA) and cancer antigen 15-3 (CA15-3) have been negative. This finding emphasizes its potential for monitoring and surveillance as a clinical indicator [265], among other areas. Broader oncology data reinforce ESM1 as a mediator in angiogenesis and tumour cell migration and further support its candidacy as a microenvironment-associated PG providing measurable readouts in clinical settings [266].

CD44, recognized for its role as an HA receptor, is also observed as an expression of GAG-modified isoforms that act as PG-like molecules. New findings have supported its involvement in BC to preserve stemness, therapy resistance, and aggressive clinicopathologic features. In HER2-amplified tumour models, residual or dormant cells that survive the HER2 blockade are enriched for CD44-positive cells, demonstrating a survival advantage and stem-like phenotype which could help to trigger tumour relapse [267]. Clinical analyses from 2023 and 2024 also connect CD44 expression with increased tumour grade, triple-negative or ER-negative, lymph node involvement and worse outcomes, suggesting that CD44 can be considered a prognostic marker and clinical indicator for cancer stem-based interventions in the breast TME [268,269].

Of all FACIT collagens, COL12A1 is emerging as a significant ECM component that has prognostic and immunologic significance in BC. In a recent study, COL12A1 was recognized as a biomarker with immune-related signature predictors of immune response [283]. Concurrently, massive transcriptomic studies have associated COL12A1 with ECM remodelling, EMT, and metastatic spread, which correspond with stromal-driven aggressive tumour progression [284].

Breast cancer-specific data for leprecan family members are limited, and definitive PG-like roles have not been defined. This gap provides an opportunity for mechanistic investigations of their glycanation patterns and interaction with binding partners to elucidate their interactions and key roles in mammary tissues. These findings, taken together, suggest that non-classified PGs (especially endocan, CD44 isoforms, and FACIT collagens such as COL12A1) are key drivers of vascular, stromal, and stem-like pathways in BC. Together, their influence on ECM structure, immune contexture, and tumour cell plasticity imply that these molecules should be included (along with canonical PGs) in the determination of microenvironment control for BC progression and relapse.

## 7. Future Perspectives

Research into PGs in BC suggests their pivotal role in regulating tumour–stroma interactions, ECM remodelling, and signalling processes in cells. Nonetheless, the nature of their structure, their subsequent PTMs, and their context-dependent roles present formidable limitations that will need to be tackled in future studies. Given that one PG can fulfil the dual functions of a tumour promoter or suppressor, depending on cellular context, BC subtype, and microenvironmental cues, one important focus would be to reveal the molecular bases of this dual role. To capture this heterogeneity and to map PG distribution and function at a high resolution, advanced methodologies such as single-cell transcriptomics, glycoproteomics, and spatial multi-omics are imperative.

Future work should also attempt to incorporate PG biology into translational and clinical contexts. Soluble PG fragments can be detected and quantified in the blood circulation which provides an opportunity for PGs to be introduced into liquid biopsy platforms and also gives rise to non-invasive biomarkers for diagnosis, early detection, and surveillance of disease progression, as well as the evaluation of treatment responses. This may be especially useful in aggressive subtypes like TNBC, where dependable biomarkers are limited. In addition, establishing PG expression signatures that are linked with therapeutic success would enable re-calibrated patient stratification and personalized patient strategies.

Continuing with this translational focus, a better understanding of how these specific PGs map to the major molecular subtypes of BC is necessary for the guidance of subtype-tailored diagnostic and therapeutic strategies. Table 4 presents an overview of PGs that are most closely associated with luminal, HER2-enriched, basal-like, claudin-low, and metaplastic tumours and features the ECM, stromal, and immune landscapes defined by the unique properties of these molecules, clustered according to clinically relevant subgroups. Thus, by incorporating these subtype-specific PG signatures, the table forms a framework that supports the development of more precise biomarker panels and stratified therapeutic approaches in BC.

From a therapeutic perspective, PGs are promising but underexploited targets. Tumour-promoting strategies aimed at inhibiting tumour-promoting PGs such as NG2, versican, or biglycan, or restoring the activity of tumour-suppressing PGs like decorin, lumican, or prolargin can reshape the TME in new ways so that they become responsive to established therapies, as well as make TME more responsive to the present anti-tumour drugs. The therapeutic approach may include novel modalities, such as monoclonal antibodies, recombinant PG fragments, or small molecules capable of modulating PG–ligand interactions to be added as adjuvants, which may supplement conventional methods in various conditions to enhance the therapeutic effect and outcome. Moreover, due to PGs contributing to immune regulation, targeting their immunomodulatory functions could enhance the efficacy of immunotherapies, an area of growing importance in BC management.

Within this context, recent progress has increased the translational potential of PGs as well as dynamic biomarkers measurable in clinical settings. PG profiling in both prospective and ongoing clinical trials is underway to demonstrate its predictable and prognostic benefits. Circulating or exosomal PGs (e.g., syndecan-1, glypican-4, and perlecan fragments) have been linked to tumour burden and therapy response in metastatic BC, which underlines their potential value as minimally invasive biomarkers for real-time disease monitoring [285,286,287]. These discoveries indicate that PG-derived molecules may act in tandem with circulating tumour DNA and exosomal RNA in multi-omic liquid biopsy panels to develop a more informative image of ECM remodelling and tumour dynamics.

In the area of therapeutics, a string of approaches targeting PG activity or expression, with potential to rewire the TME and improve therapeutic efficiency, are currently being studied. Monoclonal antibodies and small-molecule inhibitors targeting tumour-promoting PGs including chondroitin sulfate PG 4/neural/glial antigen (CSPG4/NG2), versican, or biglycan are also under study in early-phase trials [46,288], whilst recombinant PG fragments, decorin and endorepellin, demonstrated powerful anti-angiogenic and anti-metastatic activity in preclinical models. Furthermore, integrative transcriptomic data have discovered PG-related gene signatures related to BC aggressiveness and resistance to therapy, showing potential guidance for new approaches [36]. Taken together, these improvements support the position that PG modulation could synergize with conventional chemotherapy and immunotherapy to normalize the ECM and restore immune surveillance in the TME.

Similarly, building integrative bioinformatic models, which integrate glycomic, proteomic, genomic, and clinical datasets to predict PG-driven mechanisms of tumour progression and therapy resistance, is another important perspective. Systems biology approaches such as these would generate novel regulatory networks and identify actionable vulnerabilities within the TME. The use of patient-derived organoids, along with advanced 3D models that incorporate PG-rich ECM, may also provide physiologically relevant systems to test these hypotheses and accelerate the translation of basic discoveries into clinical applications.

## 8. Conclusions

PGs are pivotal regulators of BC biology through their capacity to organize the ECM and the PCM, shape ligand availability, and orchestrate cell signalling. Throughout this review, we show that PG functions emerge from the interplay between core proteins and their GAG chains, including HS, CS, DS, and KS, which together determine molecular interactions, localization, and downstream signalling outputs. In BC, these properties enable PGs to modulate adhesion, migration, invasion, angiogenesis, immune regulation within the TME, metastatic dissemination, and treatment response. Importantly, PG activity is context dependent and varies with cellular source and BC subtype, meaning that the same PG can support tumour-promoting programmes such as ECM remodelling and EMT or, conversely, restrain tumour progression by limiting pro-oncogenic signalling and preserving ECM structure.

Future PG research in BC should prioritize mechanistic and translational studies that integrate glycobiology with oncology, immunology, and computational biology to resolve PG heterogeneity at functional and structural levels. Approaches combining spatial profiling with single-cell multi-omics and detailed GAG characterization will be critical to define subtype-informed PG signatures across ER-positive disease, HER2-driven tumours, and TNBC. Ultimately, these advances should accelerate the clinical development of PG-based strategies for precision oncology, including robust biomarker panels for patient stratification and monitoring, the identification of predictors of therapeutic response, and new therapeutic interventions that target PG-mediated vulnerabilities within the TME.

## Figures and Tables

**Table 1:** Classification of PGs. Adapted from R. V. Iozzo et al. [15].

Name	Gene	Predominant GAG	Classification	Alteration in BC
Intracellular PGs				
Serglycin	*SRGN*	Hep	Secretory	Yes
Cell-surface PGs				
Syndecan 1–4	*SDC*	HS	Transmembrane	Yes
NG2	*CSPG4*	CS	Transmembrane	Yes
Betaglycan	*TGFBR3*	CS/HS	Transmembrane	Yes
Phosphacan	*PTPRZ1*	CS	Transmembrane	Yes
Glypican 1–6	*GPC*	HS	GPI-anchored	Yes
Pericellular PGs				
Perlecan	*HSPG2*	HS	BM	Yes
Agrin	*AGRN*	HS	BM	NE
COL-XV	*COL15A1*	CS/HS	BM	Yes
COL-XVIII	*COL18A1*	HS	BM	Yes
Extracellular PGs				
Aggrecan	*ACAN*	CS/KS	Hyalectan/lectican	NE
Versican	*VCAN*	CS	Hyalectan/lectican	Yes
Neurocan	*NCAN*	CS	Hyalectan/lectican	NE
Brevican	*BCAN*	CS	Hyalectan/lectican	NE
Testican 1–3	*SPOCK*	HS	Spock	NE
Canonical SLRPS				
Biglycan	*BGN*	CS	Class I	Yes
Decorin	*DCN*	DS	Class I	Yes
Asporin	*ASPN*	none	Class I	Yes
ECM2	*ECM2*	none	Class I	No
ECMX	*ECMX*	none	Class I	No
Fibromodulin	*FMOD*	KS	Class II	Yes
Lumican	*LUM*	KS	Class II	Yes
Prolargin	*PRELP*	none	Class II	NE
Keratocan	*KERA*	KS	Class II	No
Osteoadherin	*OMD*	KS	Class II	No
Epiphycan	*EPYC*	DS/CS	Class III	NE
Opticin	*OPTC*	none	Class III	No
Osteoglycin	*OGN*	none	Class III	NE
Non-canonical SLRPS				
Chondroadherin	*CHAD*	none/KS	Class IV	NE
Nyctalopin	*NYX*	none	Class IV	No
Tsukushi	*TSKU*	none	Class IV	No
Podocan	*PODN*	none	Class V	NE
Podocan-like 1	*PODNL1*	none	Class V	NE
Non-classified PGs				
Lubricin	*PRG4*	-	-	No
Endocan	*ESM1*	-	-	Yes
Leprecan	*LEPRE1*	-	-	NE
COL-IX	*COL9A1*, *COL9A2*, *COL9A3*	-	-	Yes
COL-XII	*COL12A1*	-	-	Yes
Bikunin	*AMBP*	-	-	No
CD44	*CD44*	-	-	Yes

NE: not enough evidence.

**Table 2:** Functional overview of PGs exhibiting direct pro- or anti-tumour and metastasis-related activities in BC.

Proteoglycan	Role in BC	Direct Effects on Cancer	Direct Effects on Metastasis	References
Serglycin	Tumour promoter	Promotes EMT, chemoresistance, protease activation	Enhances extravasation and metastasis	[16,17,18,19,20,21,22,23,24,25]
Syndecan-1	Tumour promoter	Angiogenesis, metabolic and inflammatory reprogramming	Pro-thrombotic, enhances dissemination	[26,27,28,29,30,31,32,33,34,35,36,37]
Syndecan-2	Tumour promoter	Invasion, migration via actin remodelling	Promotes EMT and dissemination	[38,39,40,41,42]
Syndecan-3	Tumour promoter	Hypoxia-driven metabolic adaptation	Linked to pro-metastatic microenvironment	[43,44,45,46]
Syndecan-4	Context-dependent	Regulates adhesion, angiogenesis, and VM	Reduces invasive/metastatic potential when silenced	[47,48,49,50,51]
NG2/CSPG4	Tumour promoter	Survival signalling, therapy resistance	Extravasation, metastatic niche interactions	[52,53,54,55,56,57,58,59,60,61,62,63,64,65,66]
Betaglycan	Tumour suppressor	Loss enhances TGF-β-driven progression	Loss promotes EMT and metastasis	[67,68,69,70,71,72,73,74,75,76,77,78,79,80,81]
Phosphacan	Tumour promoter	ALK/PTN signalling, chemoresistance	Associated with aggressive, metastasis-prone phenotype	[82,83,84,85,86,87,88]
Glypican-1	Tumour promoter	FGF/VEGF modulation	Promotes invasion/motility	[87,88,89,90,91,92,93,94,95]
Glypican-3	Tumour suppressor	Wnt and p38 modulation	Reduces invasion and metastasis	[96,97,98,99]
Glypican-4	Tumour suppressor	Reduced levels worsen prognosis	Circulating marker in metastatic BC	[100]
Glypican-6	Tumour promoter	NFAT and Wnt activation	Increases motility and metastatic potential	[101]
Perlecan	Context-dependent	Angiogenesis, GF gradients	Facilitates extravasation and spread	[102,103,104,105,106,107,108,109,110,111,112]
Agrin	Tumour promoter	ECM remodelling, angiogenesis	Supports metastatic immune evasion	[113,114,115,116,117,118,119]
Aggrecan	Tumour promoter	Early marker of aggressive BC	Linked to recurrence risk	[120,121,122,123,124,125,126,127,128]
Versican	Tumour promoter	Inflammation, ECM remodelling	Promotes migration, immune exclusion, and lung metastasis	[129,130,131,132,133,134,135,136,137,138,139,140]
Brevican	Tumour promoter	Modulates HA-CD44 signalling	Methylation linked to metastasis risk	[141]
SPOCK1	Tumour promoter	TGF-β signalling, EMT	Supports invasive, metastatic niches	[142,143,144,145,146,147,148,149,150,151,152,153,154,155,156,157,158]
Biglycan	Context-dependent	TLR activation, inflammation	Reactivates dormant cells, pro-metastatic niche	[159,160,161,162,163,164,165,166,167,168,169,170,171,172,173,174,175,176,177,178]
Decorin	Tumour suppressor	RTK inhibition, autophagy	Inhibits angiogenesis, metastasis	[179,180,181,182,183,184,185,186,187,188,189,190,191,192,193,194,195,196,197,198]
Asporin	Dual	TGF-β modulation, EMT	Promotes metastasis in TNBC/obesity contexts	[199,200,201,202,203,204,205,206,207]
Fibromodulin	Context-dependent	Wnt/TGF-β/NF-κB pathway	Supports metastatic ECM stiffness	[208,209,210,211,212,213,214,215,216,217]
Lumican	Dual	FAK/MAPK modulation	CAF-linked pro-metastatic remodelling	[218,219,220,221,222,223,224,225,226,227,228]
Prolargin	Tumour suppressor	ECM stability	Prevents bone metastasis	[229,230,231,232,233,234,235]
Epiphycan	Tumour promoter	TGF-β related progression	Correlates with metastatic potential	[236,237,238,239,240,241,242]
Osteoglycin	Tumour suppressor	EGFR/PI3K inhibition	Reduces metastasis, increases TILs	[243,244,245,246,247,248]
Chondroadherin	Tumour suppressor	Integrin adhesion	Peptide reduces metastasis	[249,250,251,252,253,254,255,256]
Podocan	Tumour suppressor	Cell-cycle arrest	Low levels linked to metastatic phenotypes	[257,258,259,260,261,262,263,264]
Endocan	Tumour promoter	Angiogenesis	Promotes metastatic vascularisation	[265,266]
CD44	Tumour promoter	Stemness, EMT	Migration, dissemination	[267,268,269]

**Table 3:** Comparative summary of glycosylation and sulfation characteristics among major PGs.

Proteoglycan	GAG Type(s)	Sulfation Pattern	Notes
Serglycin	CS/Hep	Highly sulfated CS chains	GAG type varies by cell context
Syndecans 1–4	HS ± CS	N- and O-sulfation	Cell-surface HSPGs modulating signalling
Glypicans 1–6	HS	N-/O-sulfated variable domains	GPI-anchored, developmental roles
Perlecan	HS	Highly sulfated HS domains	BM PG regulating gradients
Agrin	HS	Moderate sulfation	Neuromuscular and stromal roles
Versican	CS	Low–moderate CS sulfation	Splice isoforms vary in CS content
Biglycan	CS/DS	Dermatan sulfation predominant	Pro-/anti-inflammatory roles
Decorin	DS/CS	DS-rich	Strong ECM-structural roles
Asporin	None	No GAGs	GAG-lacking SLRP
Lumican	KS	Variably sulfated KS	Regulates collagen assembly
Fibromodulin	KS	KS sulfation	Stromal ECM modulator
Epiphycan	DS/CS	Moderate DS/CS sulfation	Growth-plate specific
Osteoglycin	None	No GAGs	Class III SLRP, tumour suppressive

**Table 4:** PGs most prominently associated with the major BC molecular subtypes.

BC Subtype	Most Relevant PGs	Functional Notes/Biological Relevance
Luminal A (ER^+^/PR^+^, HER2^−^, low proliferation)	Decorin, lumican, osteoglycin, glypican-3, glypican-4, and CHAD	Tumour-suppressive ECM signature; enhanced differentiation; lower invasiveness; and favourable prognosis
Luminal B (ER^+^/PR^+^, ±HER2, high proliferation)	Syndecan-1, syndecan-4, biglycan, perlecan, and versican	Promotes angiogenesis, stromal activation, TGF-β signalling, endocrine resistance, and higher tumour grade
HER2-Enriched	Syndecan-1, syndecan-4, glypican-1, agrin, and perlecan	Enhances HER2 signalling, mechanotransduction, and angiogenesis; PGs act as co-receptors to HER2 pathways
Triple-Negative/Basal-like (TNBC)	CSPG4/NG2, serglycin, versican (V1/V3/V4), SPOCK1, brevican, asporin, biglycan, and fibromodulin	Highly invasive PG profile: EMT activation, ECM degradation, immune evasion, and metabolic rewiring; poor prognosis
Claudin-Low (EMT-high, stem-like)	Versican, biglycan, asporin, and lumican (stromal)	Immune infiltration, EMT-driven biology, chronic inflammation, and stem-like microenvironment
Metaplastic BC (aggressive TNBC subtype)	CSPG4, SPOCK1, agrin, and versican	Extreme EMT phenotype, matrix stiffness, high motility, drug resistance, and metastatic progression

## Data Availability

Not applicable.

## Abbreviations

The following abbreviations are used in this manuscript:

ALK	Anaplastic lymphoma kinase
ATX	Autotaxin
BC	Breast cancer
BM	Basement membrane
BMP	Bone morphogenic protein
CAF	Cancer-associated fibroblast
CAR	Chimeric antigen receptor
CA15-3	Cancer antigen 15-3
CD44	Cluster of differentiation 44
CDKN1A	Cyclin-dependent kinase inhibitor 1A
CEA	Carcinoembryonic antigen
COL	Collagen
CS	Chondroitin sulfate
CSPG4/NG2	Chondroitin sulfate proteoglycan 4/neural/glial antigen 2
DS	Dermatan sulfate
ECM	Extracellular matrix
EGFR	Epithelial growth factor receptor
EMT	Epithelial–mesenchymal transition
EPCR	Endothelial protein C receptor
ER	Estrogen receptor
FAP	Fibroblast activation protein alpha
FGF	Fibroblast growth factor
FGF2	Fibroblast growth factor 2
GAG	Glycosaminoglycan
GPC	Glypican
GPI	Glycosylphosphatidylinositol
HA	Hyaluronic acid
Hep	Heparin
HER2	Human epidermal growth factor receptor 2
Hh	Hedgehog
HIF	Hypoxia-inducible factor
HS	Heparan sulfate
HSPG	Heparan sulfate proteoglycan
IGFR	Insulin-like growth factor receptor
IL	Interleukin
KS	Keratan sulfate
MMP	Matrix metalloproteinase
NCAM	Neural cell adhesion molecule
NFAT	Nuclear factor of activated T-cells
PCM	Pericellular matrix
PDGF	Platelet-derived growth factor
PG	Proteoglycan
PRELP	Leucine-rich repeat protein
PTM	Post-translational modification
PTN	Pleiotrophin
RTK	Receptor tyrosine kinase
SLRP	Small leucine-rich proteoglycan
SPARC	Secreted protein acidic and rich in cysteine
SPOCK	Secreted protein acidic and rich in cysteine/Osteonectin CWCV and Kazal-like domain proteoglycan
TAM	Tumour-associated macrophage
TF	Tissue factor
TFPI	Tissue factor pathway inhibitor
TGF	Transforming growth factor
TIL	Tumour-infiltrating lymphocyte
TLR	Toll-like receptor
TME	Tumour microenvironment
TNBC	Triple-negative breast cancer
TNF	Tumour necrosis factor
TSP-1	Thrombospondin-1
VEGF	Vascular endothelial growth factor
VCAN	Versican
VM	Vasculogenic mimicry
Wnt	Wingless/Integrated signaling pathway
